# Importance of suspended particulate organic matter in the diet of *Nephrops norvegicus* (Linnaeus, 1758)

**DOI:** 10.1038/s41598-020-60367-x

**Published:** 2020-02-25

**Authors:** Cesar Augusto da Silva Santana, Alina M. Wieczorek, Patricia Browne, Conor T. Graham, Anne Marie Power

**Affiliations:** 1 Ryan Institute, School of Natural Sciences, National University of Ireland Galway, University Road, Galway, Ireland; 2 Marine and Freshwater Research Centre, Galway-Mayo Institute of Technology, Dublin Road, Galway, Ireland

**Keywords:** Ecology, Stable isotope analysis, Marine biology

## Abstract

The extent to which commercially important *Nephrops norvegicus* lobsters feed on particulates in the wild is unknown, even though this could be an important way for burrow-dwelling females to avoid starvation during the long breeding season. This was investigated using δ^13^C and δ^15^N isotopic signatures in tissues with long and short turnover rates to provide diet discrimination and compare this between males and females. Secondary objectives examined size-related differences and calculated the trophic position based on the new results. Almost half the diet (47%) was made up of suspended particulate organic matter (POM_susp_) alone. Fish was another important item in the diet, with plankton and invertebrate sources coming much lower down in dietary importance. Significantly more suspension feeding was observed in small or medium sized individuals than large ones in both sexes. However, there were no sex-related patterns, despite females being restricted to burrows for part of the analysis period. Female diet was almost identical to males and POM_susp_ comprised a large component of the diet in both sexes. The trophic position was estimated at 2.94 ± 0.16 (mean ± SD), which was at the lower end of the range reported in previous studies (2.60 to 4.32).

## Introduction

Dublin Bay prawn, *Nephrops norvegicus*, is a decapod crustacean and an important economic resource in Europe: global production of this fishery was 59,033 tons in 2016 of which the United Kingdom and Republic of Ireland were the main producers, capturing up to 32,708 and 10,379 tons per annum respectively during 2012–2016^1^. *Nephrops* populations are distributed on semi-isolated mud patches which are assessed by ICES as separate Functional Units (FU), however this resource is not ‘managed’ via fishing quotas, and some FUs periodically display signs of over-exploitation^2,3^. The Marine Strategy Framework Directive and reformed Common Fisheries Policy (CFP) require ecosystem-based fisheries management which observes interactions among all components of the ecosystem, including trophic interactions^4–6^. Although not currently managed under the CFP, key gaps and ambiguities exist in knowledge of *Nephrops’* diet and feeding ecology which should be addressed, given their economic importance and occasional over-exploitation.

*Nephrops* individuals are known to be opportunistic predators and scavengers, which seem to have a diet driven by prey abundance rather than prey preference^7–10^. Diet from stomach contents analyses seems to be similar across a wide geographical range in the north-eastern Atlantic and Mediterranean, composed mainly of crustaceans, polychaetes, molluscs and echinoderms^8,9^. A considerable contribution to the diet is also made by fish in southern Atlantic and Mediterranean samples^11,12^. However, some mystery surrounds the extent of feeding on particulates in *Nephrops*. In the absence of alternative food sources, such as in the aquarium, *Nephrops* was demonstrated to feed on planktonic items larger than 300–500 µm, which were later recovered from the stomach and intestine of the animal^13^. But, although some studies use particulate organic matter (POM) as the baseline for *Nephrops* trophic position estimation^14,15^, no studies have directly measured the importance of particulate food items in the diet of *Nephrops* in its natural habitat.

The lifestyle of male and female *Nephrops* differs significantly as females are restricted to burrows while brooding embryos over a long breeding period, from six to ten months, depending on latitude^16^. Therefore, it is logical to ask whether there are sex-related differences in diet arising from these lifestyle differences. Restriction to burrows for most of the year is evidenced by a lower percentage of females in fisheries catches during the breeding season, which is elongated in Irish and Scottish grounds over Autumn, Winter and early Spring^17^. For this reason, a seasonal decrease in nutritional status, i.e. ‘starvation’, has been predicted for females in Winter, compared with late Spring and Summer when they are observed to be present in the catch and actively feeding, after releasing their broods, moulting and mating^5,10,16,18,19^. A biochemical index for estimation of nutritional status from the Clyde Sea in Scotland suggested that, although females had reduced nutritional status in the Winter, this was not sufficiently low to indicate starvation^10^. At the same time, growth in females is also much lower than in males^20^ and the respective diets of males and females are still not fully understood.

Suspension feeding has been identified as a possible strategy for females to survive starvation while they are restricted to burrows during the long breeding period^13^. Since suspended food in the form of plankton biomass is seasonally lower during the Winter female burrow-dwelling period^21,22^, we propose that females instead feed on suspended POM (i.e. POM_susp_). POM_susp_ represents a complex microscopic mixture of living and non-living organisms including phytoplankton, fecal pellets, detritus, bacteria and heterotrophs, but with distinct isotopic signatures from phyto- and zooplankton, particularly in coastal areas^23,24^, that can be an important food source to many organisms. The main aim of this study was therefore to investigate the relative importance of POM_susp_ in *Nephrops*’ diet (all sexes) as well as to examine sex-related differences in diet during the Spring-Summer period. Secondary objectives were to compare the diet composition between adult size classes as we might expect some dietary differences between the smaller and larger sizes due to differing abilities to compete over prey or to handle different prey items.

Stable isotopes analysis (SIA) was chosen to complement information from previous stomach contents analyses^8,9,11,12^. SIA can more fully represent POM_susp_ and soft-bodied prey items in the diet as well as providing a time-integrated view of feeding compared with a ‘snapshot’ provided by stomach contents analysis^25^. SIA analysis on tissues with different turnover rates can also demonstrate diet compositions over distinct periods^26^. For example, in the present study, ^13^C and ^15^N isotopic signatures in long and short-term storage tissues were used to compare signals in *Nephrops’* diet between males and females, both in the period when females were in burrows during the lead-up to spawning and the period after females had spawned and were actively feeding, maturing new gonads and mating. A final aim was to determine *Nephrops*’ trophic position based on new SIA results from the present study. The SIA data were analysed within a Bayesian framework, an approach which is increasingly used to address ecological problems^27–34^.

This study examined the importance of POM_susp_ as a food source in the diet of wild *Nephrops*, comparing this with other food sources. The specific hypotheses tested were: (i) suspension feeding is higher among the smaller (more vulnerable) adults, either because they remain in burrows to avoid enemies, or because they are too small to handle larger more mobile prey; and (ii) feeding patterns are sex-related, specifically there is higher suspension feeding in females than males during the period when females are brooding embryos and restricted to burrows compared with the period post-spawning when they are actively moulting, mating and feeding outside of the burrow.

## Results

### Importance of suspended particulate organic matter to *Nephrops*’ diet

**Figure 1:**
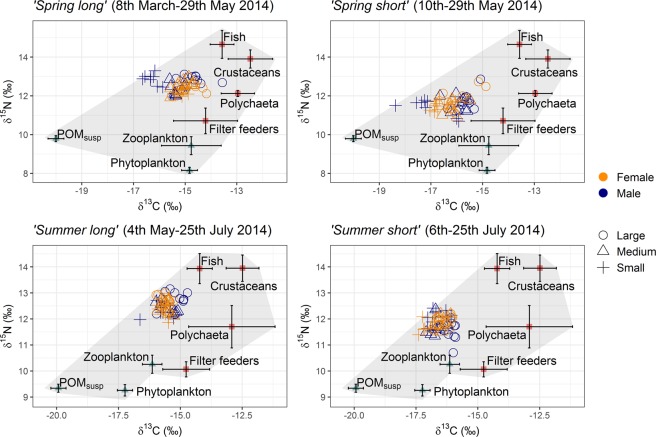
Stable isotope bi-plot of δ^13^C and δ^15^N signatures of consumers and food sources. The figure provides the isotopic signatures of *Nephrops* individuals within a polygon representing their putative prey in different time periods at Clew Bay, Ireland, 2014.

**Figure 2:**
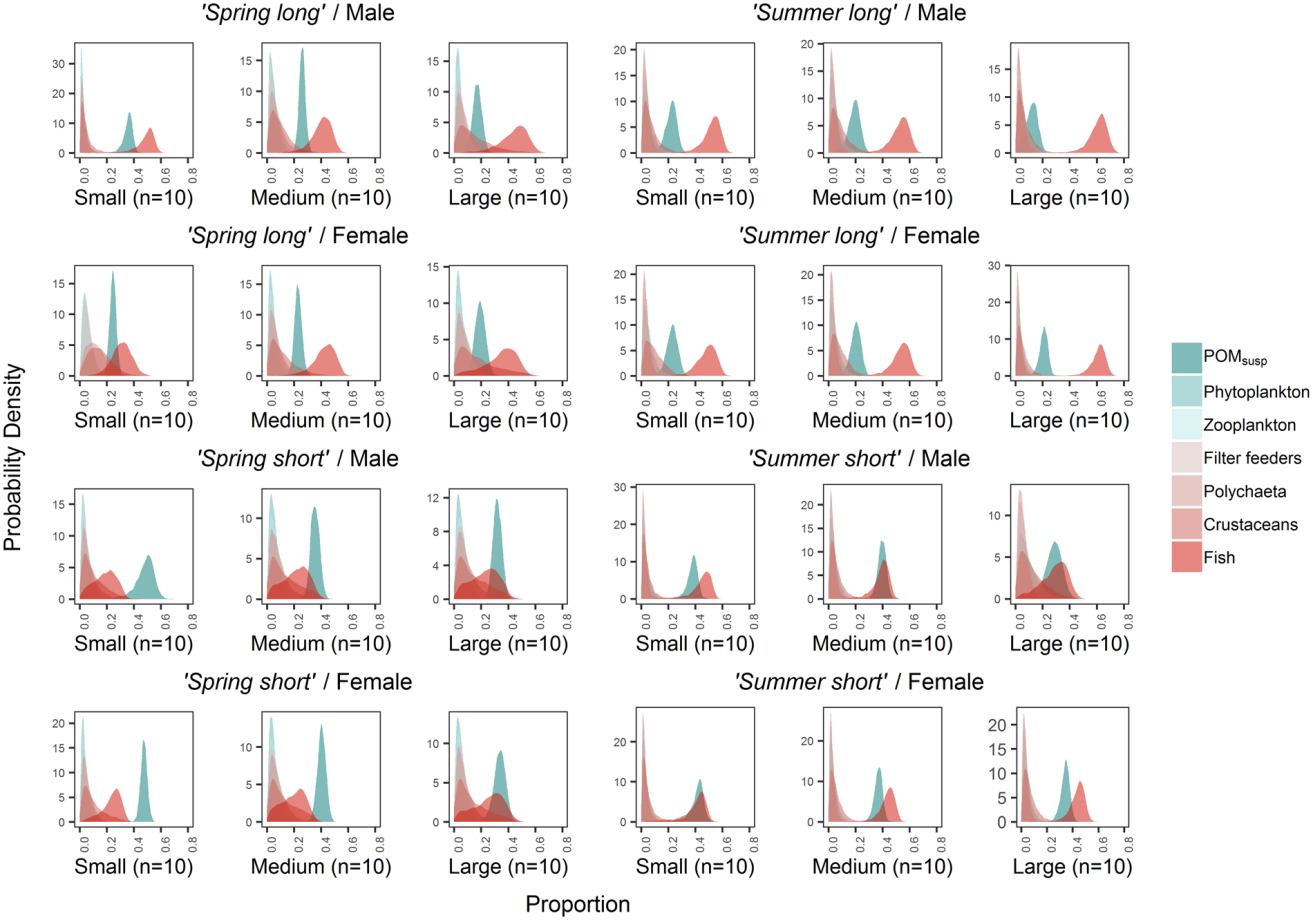
Contributions of putative sources to *Nephrops’* diet. The figure shows posterior probability distributions of the contributions of all putative sources to *Nephrops’* diet in different periods during Spring and Summer, 2014: *‘Spring long’* (8^th^ March–29^th^ May), *‘Spring short’* (10^th^–29^th^ May), *‘Summer long’* (4^th^ May–25^th^ July) and *‘Summer short’* (6^th^–25^th^ July).

Consumer isotopic values fell within the range of food source isotopic values for all four time periods (Fig. 1), indicating that all major *Nephrops*’ dietary sources were included in the analysis and no important dietary sources were missing. This arrangement between consumer and source values is a precondition for the SIMMR Bayesian mixing model to work adequately^35^. The SIMMR model output showed that POM_susp_ and fish were the main food sources for all consumer groups and time periods in the analysis. The estimated means of POM_susp_ contribution to the diet ranged between 12.0–47.4% but was generally high, >20% (Supplementary Table S1). Meanwhile, apart from fish, contributions from the other sources (phyto- and zooplankton, filter feeders, polychaetes and crustaceans) were much lower, with means ranging from 2.3–15.6% (Fig. 2). The estimated means for fish contribution to the diet ranged from 18.2–60.9%. Therefore, the main question about the importance of POM_susp_ in *Nephrops*’ diet was accepted to be the case in this study.

After combining sources, the estimated means of ‘active feeding’ (i.e. filter feeders, polychaete, crustaceans and fish) ranged from 42.5–76.2%, while that of ‘suspension feeding’ (i.e. POM_susp_, phytoplankton and zooplankton) ranged from 26.5–57.5%. More details on average contributions from all food sources and probability distributions of ‘active feeding’ and ‘suspension feeding’ to *Nephrops*’ diet can be seen respectively in Supplementary Table S1 and Fig. S1.

### Size-related differences in *Nephrops’* diet

**Figure 3:**
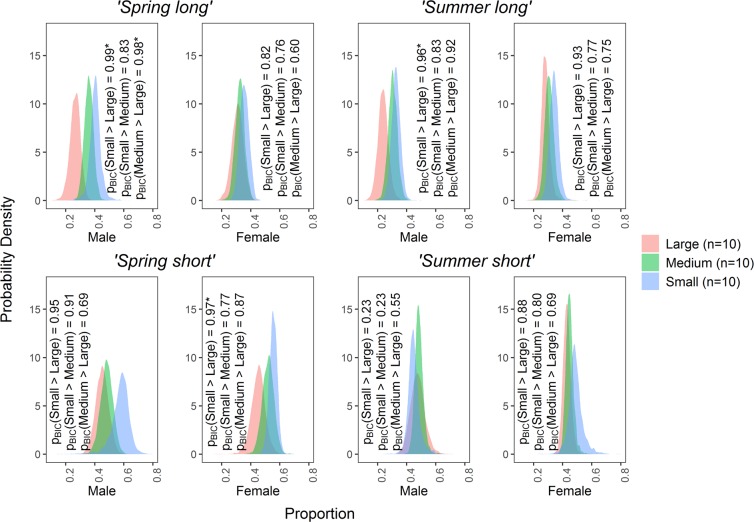
Size-related differences in *Nephrops*’ diet from suspension feeding. The figure shows posterior probability distributions comparing contribution to the diet of suspension feeding (i.e. phytoplankton + zooplankton + POM_susp_) in *Nephrops* of different sizes during Spring and Summer 2014:*‘Spring long’* (8^th^ March–29^th^ May), *‘Spring short’* (10^th^–29^th^ May), *‘Summer long’* (4^th^ May–25^th^ July) and *‘Summer short’* (6^th^–25^th^ July). An asterix indicates a significant size-related difference in suspension feeding in the diet i.e. p_BIC_ > 0.95 (provided by the SIMMR package according the Bayesian paradigm).

There were some significant *Nephrops* size-related differences in suspension feeding (i.e. POM_susp_, phytoplankton and zooplankton). The results showed that suspension feeding took place significantly more in small or medium sized individuals than large ones. Surprisingly, this occurred more frequently in male comparisons than in females. Suspension feeding was significantly higher in the small and medium sized males compared to larger males in the early time period, i.e. ‘*Spring long*’: 8^th^ March–29^th^ May (Fig. 3). In the late time period, i.e. ‘*Summer long*’ (4^th^ May–25^th^ July), it was significantly higher only in the small males compared to large males (Fig. 3). For females, the small size class was significantly more likely to suspension feed than larger ones in the ‘*Spring short’* period (10^th^–29^th^ May) (Fig. 3). No significant differences could be detected between the size groups in either sex in the *‘Summer short’* time period (6^th^–25^th^ July) (Fig. 3).

### Sex-related differences in *Nephrops’* diet

**Figure 4:**
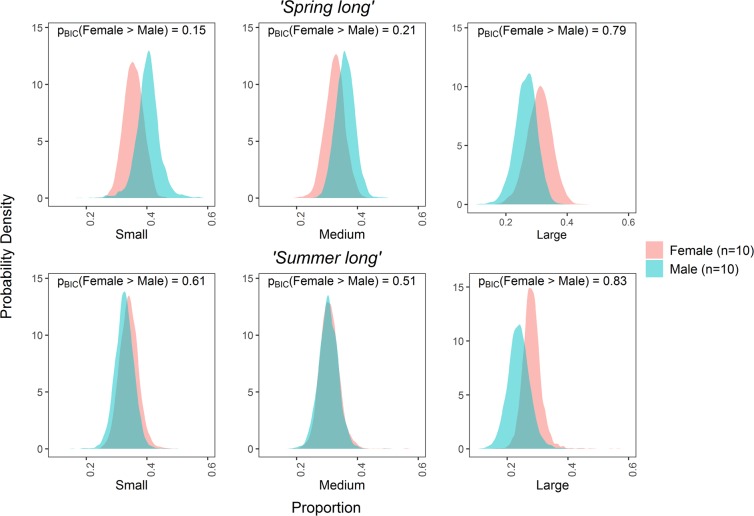
Sex-related differences in *Nephrops*’ diet from suspension feeding. The figure shows posterior probability distributions comparing contribution to the diet of suspension feeding (i.e. phytoplankton + zooplankton + POM_susp_) in *Nephrops* of different sexes, including periods when females were burrow-dwelling i.e. 8th March–29th May 2014 (*‘Spring long’*) and non-burrow dwelling periods 4th May–25th July 2014 (*‘Summer long’*). An asterix denotes a significant sex-related difference in suspension feeding in the diet i.e. p_BIC_ > 0.95 (provided by the SIMMR package according the Bayesian paradigm), however no such differences were observed.

The comparison of suspension feeding between males and females produced no significant results across time periods including: 8^th^ March–29^th^ May (*‘Spring long’*), which includes part of the long breeding season of females, and 4^th^ May–25^th^ July (*‘Summer long’*) when both sexes are non-burrow dwelling and capable of feeding outside of burrows (Fig. 4). The contribution of active feeding and suspension feeding to the diet of males and females was also equivalent, with imperceptible differences observed between the sexes (Supplementary Fig. S1). Thus, the hypothesis of differences between male and female’s diet, related to the reproductive cycle of females is not upheld at Clew Bay.

### Trophic position of *Nephrops norvegicus*

The overall trophic position of *N*. *norvegicus* in Clew Bay, based on isotopic signatures in muscle and hepatopancreas (considered together) was estimated to be 2.94 ± 0.16 (mean ± SD), which represents an average over all time periods. The trophic position across different periods varied from 2.79 ± 0.12 (*‘Spring short’*) to 3.06 ± 0.10 (*‘Spring long’*/‘*Summer long’* – with these two periods being identical; Table 1).

**Table 1:** Trophic position of *Nephrops* in Clew Bay (2014), based on δ^13^C and δ^15^N isotopic signatures from different time periods represented by long-term storage tissues (muscle: *‘Spring long’* and *‘Summer long’*) and short-term storage tissue (hepatopancreas: *‘Spring short’* and *‘Summer short’*). See Table 2 for relevant time periods.

Period	Trophic Position	
	Mean	SD
*Spring long*	3.06	±0.10
*Spring short*	2.79	±0.12
*Summer long*	3.06	±0.10
*Summer short*	2.85	±0.09
Overall (avg)	2.94	±0.16

## Discussion

The primary aim of this research was to investigate feeding on suspended particulate organic matter (POM_susp_) in wild *Nephrops* as this may be a mechanism of avoiding seasonal starvation. Before looking at its contribution to the diet, we could establish that we had captured all the important dietary sources by the arrangement of isotopic values in the δ^13^C/δ^15^N bi-plots (Fig. 1). The position of consumer tissues within the polygon of sampled food sources for each of the four periods indicated that no important food source(s) were missing in the analysis.

At times, almost half (47%) of the diet of *Nephrops* was made up of POM_susp_. These lobsters did show variety in their diet, however, and another important item in the diet was fish, while plankton and invertebrate sources came far below these items in dietary importance. Reliance on POM_susp_ and fish, rather than on invertebrates, appears initially surprising, considering predominance of invertebrates in stomach content analysis^8,9^. However, many crustaceans are predatory on fish, which is apparently independent of their size^36^. Capture of flatfish, for example, may present little difficulty to *Nephrops*, whose diet may also be subsidised from discards arising from inshore fishing activity in Clew Bay. The high level of feeding on particulate matter was more surprising, however another burrowing decapod crustacean, *Neotrypaea californiensis*, has recently been shown to be primarily reliant on POM_susp_ as a food source^33^.

Due to their burrowing lifestyle and long breeding season in females, much effort has gone into investigating seasonal starvation in *Nephrops*^10,37–39^. The ability to feed on particulate food sources would help to counteract starvation brought about by these lifestyle restrictions. When *Nephrops* individuals were maintained in unfiltered seawater in an aquarium, they showed an intermediate nutritional status between control animals with no access to food and those from the wild, which was suggestive of suspension feeding, at least *in-extremis* with no other food available^13^. The present results add to this by demonstrating the importance of suspension feeding to the diet of wild individuals, showing that they utilised this food source at a significant level. Previous work has theorised that 65–68% of daily energy intake was available for growth from suspension feeding at sufficient particulate densities^8^. Although our study does not address energy intake directly, our estimates of suspension feeding in the diet often reached 50% (particularly in short-term tissues). This likely represents a considerable amount of suspension feeding-derived energy that is available for growth (Supplementary Table S1).

In fact, it has long been acknowledged that POM_susp_ is an important seasonal source of food for benthic organisms in Winter^40,41^. Not all suspended food particles were equally important, however, for example phyto- and zooplankton were far less important than POM_susp_ in *Nephrops* (Fig. 2; Supplementary Table S1). Sediment organic matter (SOM) could be another important food source for benthic organisms like *Nephrops*. A practical difficulty is distinguishing SOM from POM_susp_ because the latter eventually falls to the seafloor and therefore forms one component of the SOM. Although we cannot discount the possibility that, as well or instead of feeding on POM_susp_, *Nephrops* also picks POM up off the sediment while deposit feeding on a mix of SOM/POM, we do not have evidence to support this idea. POM_susp_ and SOM present distinct isotopic signatures^42^. For example, compared with POM_susp_, SOM was shown to be enriched in δ^13^C and depleted in δ^15^N^42^. Had SOM with this^42^ profile been substituted for POM_susp_ in the present study, *Nephrops* samples would have fallen outside the isotopic polygon. Also, the δ^13^C/δ^15^N bi-plot in our study showed no missing dietary items, which might have been expected had SOM been important in the diet. Other studies have also shown distinct POM and SOM signatures^28,43^. Future studies combining fatty acid analysis and SIA may further disentangle the various sources of organic particulates and their relative importance, including sources found inside lobster burrows (e.g.^33^).

The hypothesis suggesting a size-related difference in *Nephrops* diet was accepted for several comparisons. Suspension feeding was higher in smaller compared to larger size classes for males in particular, e.g. during *‘Spring long’*: 8^th^ March–29^th^ May (small and medium males compared to large males) and ‘*Summer long*’: 4^th^ May–25^th^ July (small males compared to large males). Males may suffer more competition for active food items than females^44^ which may force smaller individuals to rely on particulate food sources. The same size-related difference, i.e. a higher proportion of suspended food in the diet of small females compared with larger ones, was borne out in only one time period: the *‘Spring short’* feeding on 10^th^–29^th^ May. This was not seen for the equivalent tissue later in the season ‘*Summer short*’ on 6^th^–25^th^ July. Interestingly, the size-related differences we observed did not appear to be related to a limitation on predation capacity in smaller lobsters. Indeed, at times, the contribution to the diet by fish was even higher in smaller individuals than in the larger ones, e.g. 48.1% versus 44.0% and 44.7% versus 28.1% for small versus large males in two of the four periods analysed (Fig. 2, Supplementary Table S1). However, without further research it is difficult to interpret the reason for this, for example, it is possible that smaller individuals may feed on fisheries discard or on larger individual’s leftover prey.

Although the isotopic signal from long and short-term storage tissues varied substantially, there was no difference in ‘Spring’ and ‘Summer’ diets when similar storage tissues were compared (Supplementary Fig. S1). The difference between long and short-term storage tissues arises because these represent different time intervals, 19 and 81 days respectively. Active feeding was higher in long-term storage tissues, whereas suspension feeding was increased in short-term tissues (Supplementary Fig. S1). Without further experiments, the reasons for this are unclear, however.

The hypothesis related to sex-specific diets was rejected. Males and females were remarkably similar in diet, even in the Spring period (8^th^ March–29^th^ May), even though the tissues sampled from this time represented a period when females were mostly brooding. Larval release at Clew Bay begins around the second week of April^45^, until which point, the females stay inside burrows to brood their developing embryos. Our results demonstrate that this period of burrow dwelling does not prevent females from accessing the same food items as males. It has been suggested that feeding by females during the breeding season may simply take place closer to the burrow mouth^10,46^ or females may bury food within or adjacent to burrows^15^. Although the sexes have similar diets, as shown in the present study, the overall opportunity for feeding may be reduced in females. However, starvation and sex-specific reduction in nutritional status has been previously examined and found to be absent^10^, with corroborating evidence from biochemical markers that suggest good nutritional condition throughout the year in females^38^. Although Clew Bay is a particularly shallow site, the same major food groups (i.e. plankton and particulates, macroinvertebrates and fish) are available in deeper habitats^7–12^. *Nephrops* diurnal emergence does vary with depth^47^ but we can think of no plausible reason for this to interact with the availability of POM_susp_ or other food groups. Therefore, we believe the results are transferrable to other *Nephrops* populations, although other locations may have slightly different groups of macroinvertebrates (echinoderms, in particular, were not abundant in the sediments at Clew Bay).

The importance of POM_susp_ as a dietary source in *Nephrops* has implications for its trophic position. Based on isotopic signatures in the present study, trophic level was calculated to be 2.94 ± 0.16 (mean ± SD) i.e. at the lower end of previous estimates for *Nephrops* (2.60–4.32^14,15,43^) that were also derived using SIA. SIA can reveal lower trophic status in consumers compared with stomach contents analysis, because the latter can underestimate soft-bodied prey^25^. In the case of *Nephrops*, it would be almost impossible to detect from stomach contents, the fact that up to half of the diet derived from POM_susp_. Such disproportionate measurement of prey items acts to artificially inflate trophic position based on stomach contents alone.

As the present study shows, smaller and medium-sized males fed on significantly higher suspended food than larger ones, therefore the potential to suspension feed may be an important mechanism for avoiding aggressive encounters over food between males. This is potentially important because the growth (and hence biomass) of male individuals is strongly density-dependent at Clew Bay^44^ (- densities at Clew Bay vary between 0 and 15 individuals per pot fished^45^). Body size also varies across fishing grounds - smaller *Nephrops* are found at FUs with higher stock densities, most likely as a result of reduced growth potential due to intraspecific competition^5,44^. We suggest that feeding on POM is an important lifestyle adaptation in both males (counteracting competitive interactions) and females (counteracting burrow-dwelling) but that *Nephrops* diet is remarkably similar in the sexes. The knowledge that fish is also an important component of the diet in all groups examined at Clew Bay means that, in theory, reduced subsidy from fisheries discards to scavengers like *Nephrops* under the EU Landings Obligation could affect feeding opportunities for this species in the future.

## Methods

### Study area

The research was conducted on an inshore population at Clew Bay in the west of Ireland. Samples were collected in Clew Bay (53.87°N and 9.64°W) on substrates which are dominated by mud and sand and in water depths ranging from 5–40 m (average 20 m). The tidal range is around 5 m and the residence time of water in the inner bay is likely to be short, ~2 days^48,49^. Pot fishing for *Nephrops* is seasonal in this area and runs from April – August, therefore the field sampling programme was limited to this period.

### Sample collection

All samples including *Nephrops* and putative prey (benthic macrofauna, zooplankton, phytoplankton and suspended particulate organic matter), were collected from the study site on two occasions which were eight weeks apart, i.e. on the 29-May-2014 (nominated *‘*Spring long’ or ‘Spring short*’*, depending on the tissue – see below and Table 2) and the 25-July-2014 (‘Summer long’ or ‘Summer short’ –Table 2). *Nephrops* were collected by baited creels on both dates. As *Nephrops* interacts with benthic communities both on and beneath the sediment, these potential prey items were obtained in two ways: (1) via five 15 min bottom trawl, using a standard 2 m beam trawl with a chain mat, a stretched mesh (bar length: 20 mm) and a codend liner with a knotless mesh (bar length: 4 mm) and (2) using a day grab Van Veen 12.110–250 cm²/3.14 L. A total of 17 different putative prey taxa were sampled from different groups: tunicates, polychaetes, bivalves, gastropods, crustaceans and fish on both dates (see Supplementary Table S2). Zooplankton and phytoplankton were sampled on both days using a 57 cm ring diameter and 250 µm mesh WP-2 plankton net towed behind the boat (15 min tows). The choice of plankton net assumed *Nephrops* can feed on plankton items larger than 300–500 µm^13^. Assuming that *Nephrops* consumers fell within the range of diet sources in an isotope bi-plot, we could be satisfied that no important food sources were missing from the analysis (indeed, this was the case - see Results section 2.1). To sample POM_susp_, water samples were taken via a Niskin bottle triggered at around 1 m above the seafloor. All samples were held on ice during transit and then transferred to a −20 °C freezer until processing for stable isotopes analysis.

**Table 2:** Time periods sampled based on retention time of isotopic signatures in short and long-term storage tissues in *Nephrops* consumers collected on 2 sampling days in 2014 (Supplementary Methods provide more details about residence time calculations).

Period name	Sampling day	Tissue	Residence time	Period
*Spring long*	29^th^ May	Muscle	81 days	8^th^ March–29^th^ May
*Spring short*	29^th^ May	Hepatopancreas	19 days	10^th^–29^th^ May
*Summer long*	25^th^ July	Muscle	81 days	4^th^ May–25^th^ July
*Summer short*	25^th^ July	Hepatopancreas	19 days	6^th^–25^th^ July

### Stable isotope sample preparation

Potential *Nephrops* food ‘source’ tissues were processed as follows: phytoplankton and zooplankton samples were cleaned under the microscope. POM_susp_ was concentrated by filtering seawater (around 5 L was filtered for each sample) on precombusted glass filters and stored frozen (−20 °C). POM_susp_ samples were acid-washed to remove any carbonates, which consisted of adding 1 ml 0.1 M HCl, following the protocol developed by^50^. All macrofaunal items that were dominant in both abundance and biomass in grabs were sampled for SIA using various tissues, depending on the organism (see Supplementary Table S2 for details).

‘Consumer’ (*Nephrops*) tissues were subsampled from the fisheries catch by selecting n = 10 replicate individuals within each of three size classes (small, medium and large) for both sexes (see Supplementary Table S3). After thawing at room temperature, carapace length, weight without chelipeds (to avoid bias due to claw loss) and sex was recorded for all individuals. *Nephrops* tissue was sampled from muscle (tail) and hepatopancreas for both males and females, with hepatopancreas in this case representing a shorter-term storage tissue and muscle representing a longer-term storage tissue (see below).

All tissues sampled were oven dried in 2 ml tubes at 60 °C for at least 48 h. Each dried sample was then ground with a mortar and pestle to a fine homogenous powder. Varying amounts of lipids amongst species and tissue types can result in errors in δ^13^C isotope values if not removed from the tissue prior to measurement^51^. Therefore, all source and consumer samples underwent lipid correction of three 8 ml washes (or until the supernatant was clear) of 2:1 chloroform:methanol solvent according methodology developed by^52^. Samples were again dried in the oven at 60 °C for 48 h to remove any remaining solvent. Aliquots of lipid extracted tissue of 400–600 μg were weighed into tin capsules for stable isotope analysis.

Stable isotope ratios (δ^13^C and δ^15^N) of all samples were measured at the Stable Isotope Core Laboratory of Washington State University using an elemental analyser (ECS 4010, Costech Analytical, Valencia, CA) connected to a continuous flow isotope ratio mass spectrometer (Delta PlusXP, Thermofinnigan, Bremen) and expressed as parts per thousand (‰) (further details can be found in the Supplementary Methods).

### Data analysis

The package SIMMR - Stable Isotopes Mixing Models in R^53^ was used to estimate the likely contribution of each putative food source to the diet of *Nephrops* by solving mixing equations for stable isotopic data within a Bayesian framework. SIMMR model outputs are posterior probability distributions representing the likelihood of a specific source being part of the diet of the consumer, with their respective credible intervals. SIMMR was run based on the following input data: ^13^C and ^15^N isotope signatures of consumers, mean ^13^C and ^15^N isotope signatures of sources i.e. putative prey groups and their standard deviations and estimates for ^13^C and ^15^N trophic enrichment factors (means and standard deviations – see below).

For the initial analysis, to show the importance of POM_susp_ in the diet and to ensure that all dietary sources were captured in the analysis, sources were divided into seven taxa/groupings: (i) Crustaceans; (ii) Filter feeders; (iii) Fish; (iv) Phytoplankton; (v) Polychaeta; (vi) POM_susp_ and (vii) Zooplankton. Meanwhile, consumers were grouped in all possible combinations of size (small, medium, large), sex (male and female) for long/short-term storage tissues (respectively, muscle and hepatopancreas), providing 12 different combinations overall. Comparison of diet between these consumer groups formed the basis of further hypothesis testing, i.e. statistical comparisons of ‘active feeding’ versus ‘suspension feeding’, as described in ‘Statistical design’, below. The SIMMR model was run twice based on isotopic signatures (for both consumers and food sources) collected in each of the first and second sampling days. Next, four experimental time ‘Periods’ were defined based on the combination of the two sampling dates and two different tissues representing a long (muscle) or short (hepatopancreas) residence times (*rt*) (Table 2). Residence time for muscle tissue was 81.1 days, obtained from isotopic incorporation rates and discrimination factors in *Neogonodactylus bredini* (mantis shrimp)^54^, while *rt* for hepatopancreas was estimated as 19.3 days. This was calculated from the ^13^C half-life for hepatopancreas tissues in *Callinectes sapidus*^55^ (further details of these calculations can be found in the Supplementary Methods).

Trophic enrichment (or ‘fractionation’) factors (TEFs) of 3.0 ± 0.6‰ for δ^13^C and 0.9 ± 0.3‰ for δ^15^N were chosen, based on estimates from mantis shrimp muscle^54^, which is the best taxon-specific information available. These values contrast with widely-used values from previous meta-analysis^56^ that present averages from 61 different species of aquatic and terrestrial vertebrates and invertebrates in a variety of taxa: arthropods, molluscs, nematodes, birds, fish and mammals (for information, values in^56^ were 0.5 ± 0.13‰ for δ^13^C and 2.3 ± 0.18‰ for δ^15^N). Nevertheless, we chose the mantis shrimp values^54^, firstly, on the basis that these fractionation values were calculated from a decapod crustacean: taxonomic relatedness is important due to evidence that TEFs are taxon-specific due to shared physiological processes at taxon level^57–61^. Secondly, the values in^54^ represented lipid-corrected stable isotope ratios for consumers and prey, as also used in our study, and were from a diet shift controlled laboratory experiment.

### Statistical design

Each group of consumers subjected to hypothesis testing included 10 replicate consumer samples (n = 10). This sample size seems adequate in bootstrapped simulations, which have shown an absence of large biases in statistical inference of stable isotope data with >8 replicate consumer samples^62^. For hypothesis testing, sources were combined by the function ‘*combine_sources’* of SIMMR package into ‘active feeding’ (i.e. filter feeders, polychaete, crustaceans and fish) and ‘suspension feeding’ (i.e. POM_susp_, phytoplankton and zooplankton). In order to test our hypotheses, posterior distributions of ‘suspension feeding’ by consumers were compared in several ways. Size-related differences in consumers were examined across all 8 possible *‘*Sex’ x ‘Period’ combinations (i.e. all combinations of males and females in 4 time periods). Sex-related differences in consumers were examined across 6 combinations of 3 ‘Size’ groups in 2 periods, *‘Spring long’* and *‘Summer long’*. These periods represent an equivalent number of feeding days but with the key difference that *‘Spring long’* included part of the period where females were brooding embryos in Clew Bay, i.e. up until ~10^th^ April^45^, whereas *‘Summer long’* was a non-brooding period. Any dietary differences associated with female brooding could be judged against males using this comparison. Please note that, as they had just completed their reproductive cycle and had spawned, none of the females sampled actually contained embryo masses, however we could assume that 84–92% of our sample (n = 60) of females had bred, based on previous work^17,63–65^. The suspension feeding contribution was compared across each of the above groups using the function *‘compare_ groups’* from the SIMMR package. This function gives the probability *p*_BIC_ of ‘any diet source’s proportion in one treatment being greater than the proportion of the same source in another treatment’ with *p*_BIC_ > 0.95 considered to indicate significant differences^66^.

The trophic position of *Nephrops* was determined based on the isotopic signatures of consumers and prey according to a modified version of the following equation^67^:

1 $$TP=\lambda +({\delta }^{15}{N}_{c}-{\delta }^{15}{N}_{base})/{\Delta }_{n}$$

where ${\delta }^{15}{N}_{c}$ is the isotopic signature of the consumer *Nephrops*, *N*_*base*_ is that of the food base (herein phytoplankton), λ is the trophic position of the base (λ = 1 for primary producers) and Δ_*n*_ is an estimate of the average increase in *Δ*^15^*N* per trophic position/level, herein set at 3.4‰ based on estimates for aquatic food webs^67,68^. However, because the TEF for δ^15^N, is significantly lower for decapods^54^ than for many other taxa^56,67,68^, we modified the Eq. (1) to incorporate this and prevent an erroneous underestimation of *Nephrops’* trophic position, as follows:

2 $$TP=1+(\lambda +(({\delta }^{15}{N}_{c}-0.9)-{\delta }^{15}{N}_{base})/{\Delta }_{n})$$

where 0.9 is the TEF for δ^15^N of mantis shrimp^54^ corresponding to the average increase in *Δ*^15^*N* per trophic position/level; this was subtracted from the isotopic signatures of the consumers in Eq. (1) to facilitate a more accurate trophic position calculation for a decapod, as is the case in the present study. Because this manipulation of the equation underestimates the trophic position in one level, a correction was required by adding one trophic position/level at the end of the calculation, as seen in Eq. (2).

For estimating *Nephrops*’ overall trophic position, the isotopic signatures in the tissues (muscle and hepatopancreas together) of all individuals sampled in both sampling days (n = 120) were used. In this case, the trophic position of each individual was estimated, and the average of these values was considered to represent the trophic position of *Nephrops* in Clew Bay in 2014. The average of the nitrogen isotopic signatures of the phytoplankton collected in each of the 2 sampling days was used as $\,{\delta }^{15}{N}_{base}$.

## Supplementary information

Supplementary information.

## Data Availability

Supplementary Information accompanies this paper. The datasets generated during and/or analysed during the current study are available from the corresponding author on reasonable request.
