# Supplementary material for: Importance of suspended particulate organic matter in the diet of Nephrops norvegicus (Linnaeus, 1758)
Source: Sci Rep. 2020 Feb 25;10:3387. doi: 10.1038/s41598-020-60367-x (PMC7042260; doi:10.1038/s41598-020-60367-x)
Supplement: Supplementary file 1 — Supplementary information. [file 41598_2020_60367_MOESM1_ESM.pdf]

Scientific Reports

Supplementary Material

Importance of suspended particulate organic matter in the diet of *Nephrops norvegicus* (Linnaeus, 1758)

Cesar Augusto da Silva Santana[a], Alina M. Wieczorek[a], Patricia Browne[a], Conor T. Graham[b], Anne Marie Power[a*]

[a]*Ryan Institute, School of Natural Sciences, National University of Ireland Galway, University Road, Galway, Ireland*

[b]*Marine and Freshwater Research Centre, Galway-Mayo Institute of Technology, Dublin Road, Galway, Ireland*

*Corresponding author: Tel: +353-91-493015; e-mail: annemarie.power@nuigalway.ie

Supplementary Table S1. Average contributions of the main food sources to *Nephrops* diet at Clew Bay, in different periods: *'Spring long'* (8th March-29th May 2014), *'Spring short'* (10th -29th May 2014), *'Summer long'* (4th May-25th July 2014) and *'Summer short'* (6th-25th July 2014). POM = suspended Particulate Organic Matter.

Spring long	Male			Female		
	Small	Medium	Large	Small	Medium	Large
POM	35.3%	26.1%	16.8%	24.5%	22.9%	19.4%
Phytoplankton	2.3%	4.8%	4.3%	5.5%	4.2%	5.3%
Zooplankton	2.8%	5.2%	5.4%	5.5%	5.3%	6.5%
**Suspension feeding**	**40.4%**	**36.1%**	**26.5%**	**35.5%**	**32.4%**	**31.2%**
Filter feeders	3.1%	5.9%	6.4%	6.2%	6.1%	7.5%
Polychaetes	3.4%	7.4%	7.5%	12.2%	7.1%	8.7%
Crustaceans	5.0%	10.4%	15.6%	15.2%	12.3%	18.4%
Fish	48.1%	40.2%	44.0%	30.9%	42.1%	34.2%
**Active feeding**	**59.6%**	**63.9%**	**73.5%**	**64.5%**	**67.6%**	**68.8%**

Spring short	Male			Female		
	Small	Medium	Large	Small	Medium	Large
POM	47.4%	35.7%	32.0%	47.4%	40.5%	33.5%
Phytoplankton	4.6%	5.6%	5.9%	3.6%	5.2%	5.4%
Zooplankton	5.5%	6.8%	7.4%	4.4%	6.3%	6.4%
**Suspension feeding**	**57.5%**	**48.1%**	**45.3%**	**55.4%**	**52.0%**	**45.3%**
Filter feeders	6.6%	8.1%	8.6%	5.2%	7.6%	7.5%
Polychaetes	7.1%	8.7%	9.0%	5.7%	8.3%	8.4%
Crustaceans	10.6%	14.1%	14.6%	10.6%	13.1%	13.6%
Fish	18.2%	21.0%	22.5%	23.1%	19.0%	25.2%
**Active feeding**	**42.5%**	**51.9%**	**54.7%**	**44.6%**	**48.0%**	**54.7%**

Summer long	Male			Female		
	Small	Medium	Large	Small	Medium	Large
POM	21.9%	19.1%	12.0%	22.7%	20.0%	20.6%
Phytoplankton	5.3%	5.5%	5.9%	5.5%	5.2%	3.7%
Zooplankton	5.4%	5.9%	5.9%	5.9%	5.7%	3.8%
**Suspension feeding**	**32.6%**	**30.5%**	**23.8%**	**34.1%**	**30.9%**	**28.1%**
Filter feeders	3.9%	4.0%	4.2%	3.9%	3.8%	2.7%
Polychaetes	3.9%	4.1%	4.2%	3.7%	3.5%	2.7%
Crustaceans	7.6%	8.6%	7.0%	9.9%	8.5%	5.6%
Fish	52.0%	52.8%	60.8%	48.4%	53.3%	60.9%
**Active feeding**	**67.4%**	**69.5%**	**76.2%**	**65.9%**	**69.1%**	**71.9%**

Summer short	Male			Female		
	Small	Medium	Large	Small	Medium	Large
POM	37.7%	39.6%	28.5%	40.7%	37.0%	34.1%
Phytoplankton	3.6%	4.4%	9.5%	3.9%	3.7%	4.3%
Zooplankton	3.7%	4.5%	9.8%	4.1%	4.0%	4.5%
**Suspension feeding**	**45.0%**	**48.5%**	**47.8%**	**48.7%**	**44.7%**	**42.9%**
Filter feeders	2.7%	3.3%	6.2%	3.0%	2.9%	3.3%
Polychaetes	2.8%	3.3%	5.5%	3.2%	3.0%	3.4%
Crustaceans	4.8%	6.1%	12.4%	5.4%	5.8%	6.8%
Fish	44.7%	38.8%	28.1%	39.7%	43.6%	43.6%
**Active feeding**	**55.0%**	**51.5%**	**52.2%**	**51.3%**	**55.3%**	**57.1%**

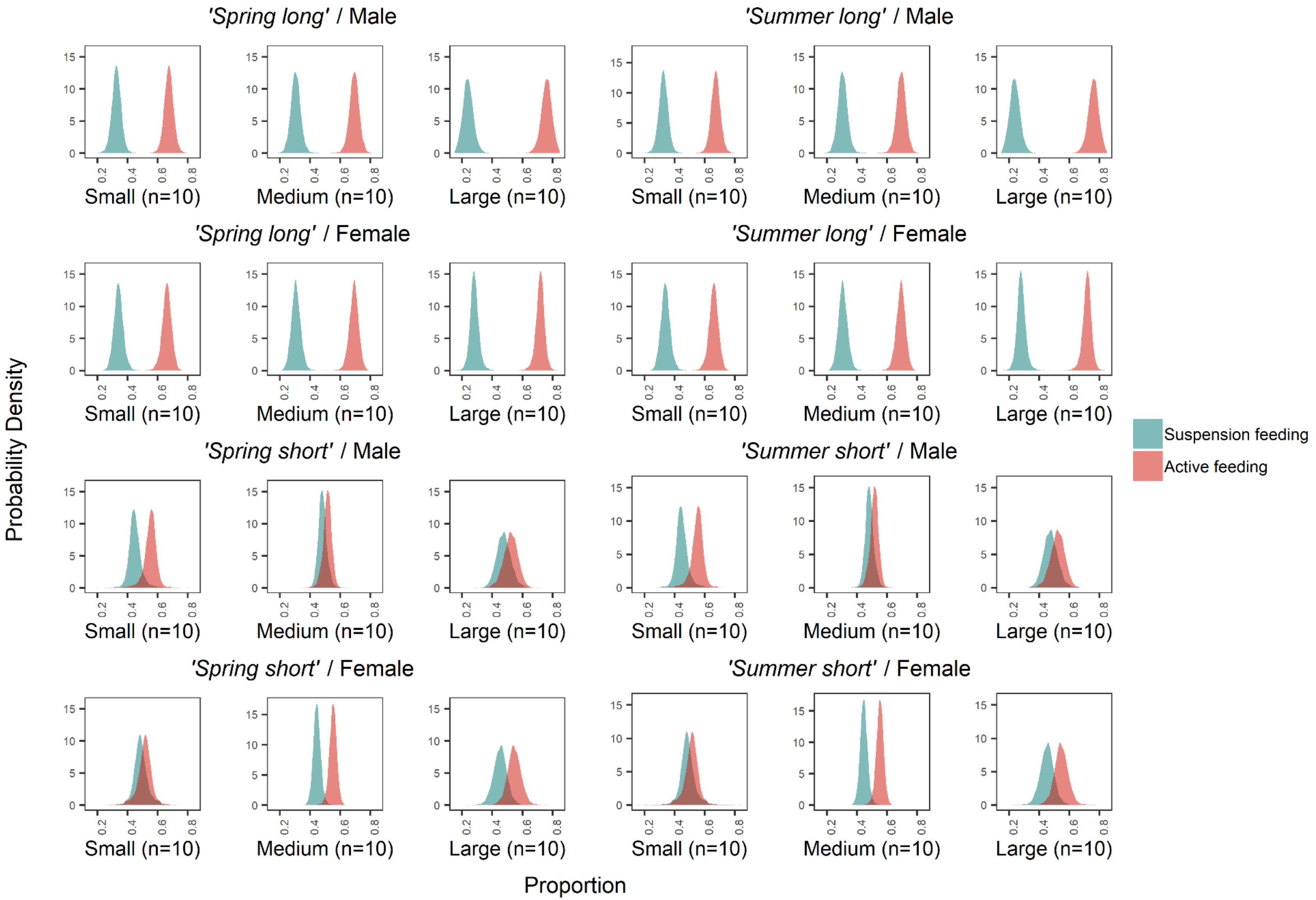

Supplementary Figure S1. Probability distributions of the contributions of active and suspension feeding to the diet of *Nephrops* in different periods during Spring and Summer 2014: *'Spring long'* (8th March - 29th May 2014), *'Spring short'* (10th - 29th May 2014), *'Summer long'* (4th May - 25th July 2014) and *'Summer short'* (6th - 25th July 2014).

Supplementary Table S2. Putative macrofaunal prey species and tissue type analysed for stable-isotope ratios, along with mean isotopic signatures.

Group	Species	Nº samples	Tissue type analysed	Group mean ± SD $\delta^{13}C$	$\delta^{15}N$
**Sampling date 1: 29-May-2014**					
**Filter feeders**	*Turritella communis*	3	Foot muscle	**-17.15 ± 1.19**	**9.89 ± 0.70**
	Aequipecten opercularis	4	Adductor muscle		
	Tunicate sp.	2	Whole body		
	Terebellidae sp.	1	Whole body		
	Pecten maximus	4	Adductor muscle		
	Ostrea edulis	4	Adductor muscle		
**Polychaete**	*Nephtydiae sp.*	4	Whole body	**-15.90 ± 0.64**	**11.24 ± 0.03**
**Crustaceans**	*Liocarcinus depurator*	4	Cheliped muscle	**-15.47 ± 0.80**	**13.00 ± 0.44**
	Pagurus bernhardus	4	Cheliped muscle		
	Necora puber	4	Cheliped muscle		
	Carcinus maenas	4	Cheliped muscle		
	Palaemon serratus	4	Cheliped muscle		
	Crangon crangon	4	Cheliped muscle		
	Cancer pagurus	1	Cheliped muscle		
**Fish**	*Trisopterus minutus*	1	Dorsal muscle	**-16.57 ± 0.34**	**13.75 ± 0.70**
	H. platessoides	1	Dorsal muscle		
	Limanda limanda	4	Dorsal muscle		
	Callionymus lyra	4	Dorsal muscle		
	Merlangius merlangus	4	Dorsal muscle		
**Sampling date 2: 25-July-2014**					
**Filter feeders**	*Turritella communis*	3	Foot muscle	**-17.85 ± 0.84**	**9.35 ± 0.47**
	Aequipecten opercularis	4	Adductor muscle		
	Tunicate sp.	2	Whole body		
	Terebellidae sp.	3	Whole body		
	Anomia ephippium	4	Adductor muscle		
**Polychaete**	*Nephtydiae sp.*	4	Whole body	**-14.58 ± 0.22**	**11.28 ± 0.72**
**Crustaceans**	*Liocarcinus depurator*	4	Cheliped muscle	**-15.49 ± 0.59**	**13.05 ± 0.48**
	Pagurus bernhardus	4	Cheliped muscle		
	Necora puber	4	Cheliped muscle		
	Carcinus maenas	4	Cheliped muscle		
	Palaemon serratus	4	Cheliped muscle		
	Crangon crangon	4	Cheliped muscle		
	Cancer pagurus	4	Cheliped muscle		
**Fish**	*Trisopterus minutus*	4	Dorsal muscle	**-17.22 ± 0.41**	**13.03 ± 0.56**
	H. platessoides	4	Dorsal muscle		
	Merlangius merlangus	4	Dorsal muscle		
	Callionymus lyra	4	Dorsal muscle		

Supplementary Table S3. *Nephrops* size classes of males and females sampled on 29th May and 25th July 2014 for stable isotope analysis. Size classes were defined based on the range of carapace lengths of the samples (27.30-58.10 mm). Small individuals were defined as the ones with CL ≤ 36 mm, large individuals were the ones with CL ≥ 44 mm and medium were the ones with CL between these values (36-44 mm).

Sampling date	Sex	Size class	Range of Carapace length (mm)
**29-May-2014**	Male	Small	27.30-34.00
		Medium	37.30-43.70
		Large	45.20-58.10
	Female	Small	30.70-35.30
		Medium	39.00-43.30
		Large	44.60-50.40
**25-July-2014**	Male	Small	27.50-35.80
		Medium	38.40-41.40
		Large	48.20-56.50
	Female	Small	27.70-33.70
		Medium	36.40-42.70
		Large	44.00-55.10

Supplementary Methods

Further details about stable isotope analysis

Isotope analysis was carried out at carried out at the Stable Isotope Core Laboratory of Washington State University (see also main text). The samples were converted into N_2 and CO_2 and separated with a 3 m gas chromatography (GC) column connected to a continuous flow isotope ratio mass spectrometer (Delta PlusXP, Thermofinnigan, Bremen) and Stable Isotope Ratios (R) were expressed in δ notations as parts per thousand (‰) using the following equation:

$$\delta X = \left(R_{\text{sample}}/R_{\text{standard}} - 1\right) x1000 \qquad \text{Eq. (1)}$$

The internationally accepted standards for carbon and nitrogen were: Vienna Pee Dee Belemite and atmospheric nitrogen, respectively. Samples were normalised through internal running standards (acetanilide and keratin), which were previously calibrated using sucrose reference material and was shown to be precise (mean ± SD: - 26.92 ± 0.07 for $\delta^{13}C$ and 6.04 ± 0.12 for $\delta^{15}N$).

Further details about residence time (*rt*) estimates

Sometimes isotopic residence times (= 'rt') for different tissues can be obtained directly from the literature. For example, the *rt* of the ^{13}C and ^{15}N isotope signatures in muscle tissue of *Nephrops* was estimated from mantis shrimp[2] (also a decapod) and defined to be 81.05 days, which was the mean of the *rt* values for ^{13}C and ^{15}N residence times in that study. When literature provides only estimates of values for isotopic half-lives ($t_{1/2}$) in the tissue or tissue-specific turnover rates (λ), rt can be estimated, by the following equations[1]:

$$rt = t_{1/2}/ln2 \qquad \text{Eq. (2)}$$

$$rt = 1/\lambda \qquad \text{Eq. (3)}$$

As the mantis shrimp study[2] used above only considered muscle and haemolymph tissues, the value for the residence time of *Nephrops* hepatopancreas tissue was estimated from the ^{13}C half-life of *Callinectes sapidus* (blue crab)[3] i.e. a half-life of 13.4 days. From this value, a residence time of 19.3 days could be derived from Eq. 2 above and, as they are both decapods, this value was also used for residence time of *Nephrops* hepatopancreas tissue. Finally, as there were two tissue types and two sampling days, four time-related sampling intervals were defined (see Table 1 of main text).

Supplementary References

1. Thomas, S. M., Crowther, T. W. Predicting rates of isotopic turnover across the animal kingdom: a synthesis of existing data. *J. Anim. Ecol.* **84,** 861-870 (2015).

2. deVries, M. S., del Rio, C. M., Tunstall, T. S., Dawson, T. E. Isotopic incorporation rates and discrimination factors in mantis shrimp crustaceans. *PLoS One* **10(4),** 1-16 (2015).

3. Vedral, A. J. Blue crab residency and migration in the Mobile Bay estuary: a stable isotope study investigating connectivity (PhD Thesis). University of Alabama (2012). Available at: http://acumen.lib.ua.edu/content/u0015/0000001/0001038/u0015_0000001_0001038.pdf (Accessed: 01/09/2019).
